# Detecting Steps Walking at very Low Speeds Combining Outlier Detection, Transition Matrices and Autoencoders from Acceleration Patterns

**DOI:** 10.3390/s17102274

**Published:** 2017-10-05

**Authors:** Mario Muñoz-Organero, Ramona Ruiz-Blázquez

**Affiliations:** 1 Telematics Engineering Department, Carlos III University of Madrid, 28903 Getafe, Spain; raruizb@it.uc3m.es; 2 UC3M-BS Institute of Financial Big Data, Carlos III University of Madrid, 28903 Getafe, Spain

**Keywords:** step detection, machine learning, outlier detection, transition matrices, autoencoders

## Abstract

In this paper, we develop and validate a new algorithm to detect steps while walking at speeds between 30 and 40 steps per minute based on the data sensed from a single tri-axial accelerometer. The algorithm concatenates three consecutive phases. First, an outlier detection is performed on the sensed data based on the Mahalanobis distance to pre-detect candidate points in the acceleration time series that may contain a ground contact segment of data while walking. Second, the acceleration segment around the pre-detected point is used to calculate the transition matrix in order to capture the time dependencies. Finally, autoencoders, trained with data segments containing ground contact transition matrices from acceleration series from labeled steps are used to reconstruct the computed transition matrices at each pre-detected point. A similarity index is used to assess if the pre-selected point contains a true step in the 30–40 steps per minute speed range. Our experimental results, based on a database from three different participants performing similar activities to the target one, are able to achieve a recall = 0.88 with precision = 0.50 improving the results when directly applying the autoencoders to acceleration patterns (recall = 0.77 with precision = 0.50).

## 1. Introduction

The automatic recognition of human activities and movements using wearable sensor data is able to provide contextual information to many areas of application. Some examples are ambient-assisted living [1], the self-management and monitoring of health parameters for patients with chronic conditions [2], training in sports [3], or entertainment and security [4]. By using the data extracted from sensors such as accelerometers, physiological sensors, Global Position System (GPS), or environmental sensors, several movement related features can be computed and machine learning algorithms can be trained to classify different activities and movements [4].

One particular application of using sensor data to detect human movements is for counting steps while walking at very slow speeds. People with physical disabilities or medical long-term conditions may find it difficult to walk at normal speeds [5]. However, physical activity is normally recommended to this particular set of users in order to promote or regain a healthy lifestyle. Counting steps is a supporting mechanism for accurately measuring physical activity for these users. Among the available wearable sensors, accelerometers and pedometers are commonly used for tracking ambulatory physical activity in clinical populations. By using the sensor display, information such as the number of steps walked and/or the number of calories burnt could be shown to the user, which are useful in motivating patients to increase their activity levels [5]. However, the accuracy of accelerometers for gait analysis tends to decrease (underestimating the real number of steps) in slow walking conditions [6]. Some examples of of-the-shelf sensors and devices that underestimate strides at slower walking speeds can be found in [7]. The authors in [7] found that the number of steps counted by two off-the shelf devices while walking at a walking cadence around 67 steps/minute was around 90% of the real value. This degradation would increase for slower cadences.

In this paper, we propose a novel mechanism to detect and count steps at slow speeds by processing acceleration data from a wearable device. We validate the proposed algorithm by using the data from three different participants executing slow-walking segments in the middle of several related activities such as sliding (walking without lifting the feet from the ground) at slow speeds, walking in circles at slow speeds, or walking at moderate speeds. The algorithm combines outlier detection for pre-selecting and aligning candidate steps, transition matrices to capture time dependences in the sensed time series, and autoencoders to assess the similarity of pre-selected segments with real steps used for training. Outlier detection from acceleration time series will identify segments of interest that exhibit particular stochastic properties, such as the ground contact instant while walking.

## 2. Related Work

Physical and mental health conditions benefit from physical activity. A healthy lifestyle improves both physical and mental health aspects [8]. In order to monitor the physical activity of a particular user, the counting of steps is one of the most used measures, and therefore it is important that activity-monitoring devices are both specific and sensitive in estimating the actual number of steps walked (discriminating real steps from non-stepping body movements) [8]. Many off-the-shelf devices register a significant number of false positive steps per minute when executing non-walking activities [8] and fail to count steps while walking at slow speeds [7]. Some previous studies have reviewed the accuracy of accelerometers for gait analysis in slow walking, such as [6,7,9,10], finding that off-the-shelf sensors tend to underestimate strides (significantly in some cases) at slower walking speeds (more specifically for patients of severe medical conditions which affect the gait) [11].

Different approaches and algorithms have been previously used for counting steps in different scenarios. The authors in [12] used a threshold based algorithm, characterized by a linear computational time, trying to improve the real-time monitoring and real-time analysis of the walking behaviour of animals such as dairy cows. The research in [13] proposed an algorithm based on the use of an Android smartphone accelerometer, Fast Fourier Transform (FFT), and thresholding for detecting steps. The algorithm was not validated for slow walking and achieved poor results for running segments of data. The study in [14] used a wrist worn accelerometer to estimate the walking cadence and speed in daily life walking in several environments. The study in [15] proposed the use of some particular points in the acceleration time series in order to detect steps at normal speeds.

There are some preliminary results for detecting steps while walking at slow speeds based on raw acceleration data [6,16]. The slow execution of movements can contribute to increasing the difficulty in the process of detecting steps from acceleration data and in order to minimize the false positives from other activities.

Outlier detection has attracted a significant interest in several different areas. The authors in [17] applied outlier detection to estimate peak ground accelerations in seismic data. An algorithm for collision and hazard detection for motorcycles via inertial measurements based on outlier detection was presented in [18]. The authors in [19] made use of univariate outlier detection techniques in order to detect unusual sleep patterns. The authors in [20] used a density-based outlier detection method by measuring the LOF (Local Outlier Factor) on a projected PCA (Principal Component Analysis) domain from real world spatiotemporal traffic signals to detect traffic data outliers, which are errors in data and traffic anomalies in real situations, such as accidents and congestions. The research in [21] also used an outlier detection algorithm in a traffic system as a basis for alerting the transport department and drivers about some abnormal traffic conditions, such as traffic accidents or traffic congestion.

Outlier detection has also been previously used for the recognition of human movements. By using different sensor technologies, some human movements can be studied in terms of sequential data analysis. The authors in [22] used models that utilize sequential data as a measure to determine if the executions of a particular activity are close enough to a pre-defined specification of the activity or if they should be considered as executed in a wrong way. The authors used Hidden Markov Models (HMM) for time series characterization. The research study in [23] tackled the problem of fall detection by using a combination of outlier detectors. Using HMM for outlier detection and its application to fall detection has been studied in [24].

The research in this paper combines outlier detection from acceleration time series with time dependencies characterization via a transition matrix to feed a detection algorithm based on the similarity between the input and output of an autoencoder. Data from real steps while walking at slow speeds are used to train the autoencoder. The trained autoencoder is used to reconstruct the transition matrices at pre-selected points based on a Mahalanobis distance outlier detection mechanism. The Pearson correlation index is used to assess the similarity between the input and output of the autoencoder in order to determine if the pre-selected point is a step at slow speed walking.

The rest of the paper is organized as follows. Section 3 is dedicated to presenting the mechanism used to obtain the input data from the acceleration time series. Section 4 describes the outlier detection algorithm used in this paper. Section 5 is dedicated to present the mechanism used to estimate the transition matrices. The way we use autoencoders is shown in Section 6. Section 7 details the experimental results and Section 8 captures the main conclusions of this research.

## 3. Sensor and Data Series

Smart mobile devices such as smartphones or tablets contain several sensors that are able to automatically monitor and track some user related information. In our case, the proposed algorithm in this paper is based on the use of the accelerometer and gravity sensors. Combing the output of these sensors, we obtain acceleration time series that are both gravity free and geo-referenced in order to characterize the user’s movements.

**Figure 1:**
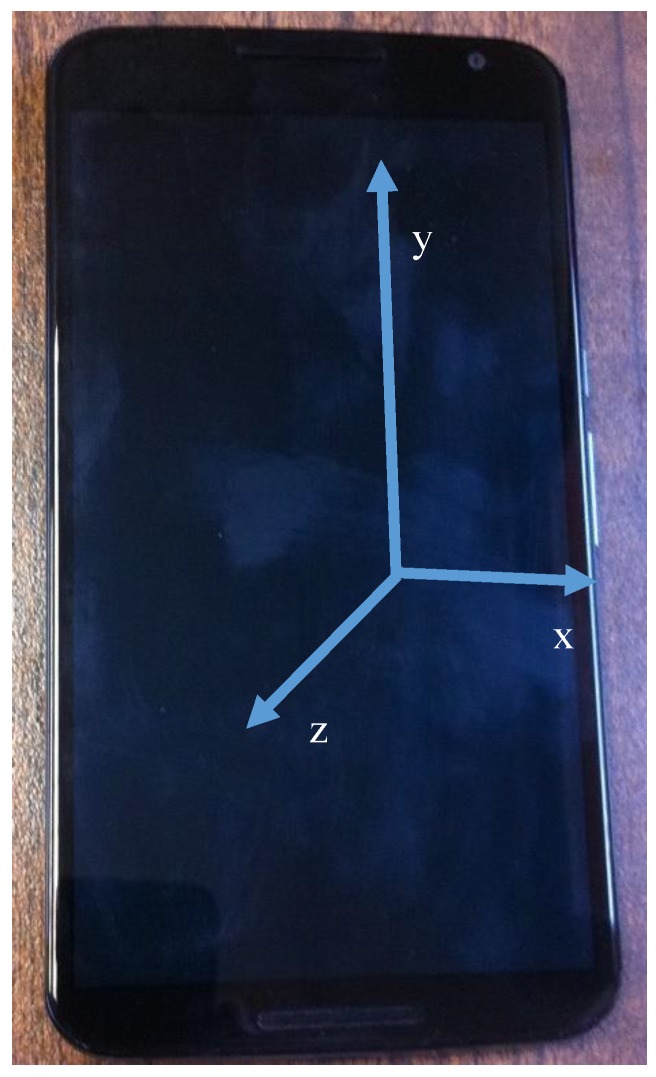
Nexus 6 mobile device and accelerometer axes.

Figure 1 shows the device used to record the raw acceleration data and the three axes as defined by the Android operating system to provide accelerometer and gravity information. The device used was a Nexus 6 Android mobile device, which contains an accelerometer sensor and is able to estimate the gravity force vector using the combination of the accelerometer, gyroscope, and magnetic field sensors. The device is able to obtain 50 samples per second from these two sensors.

The data gathering process performs a series of steps. First, we remove the gravity component from the acceleration time series provided by the accelerometer sensor by subtracting the output from the gravity sensor to the accelerometer sensor output, as captured in Equation (1). $\vec{a}$ represents the gravity free acceleration, $\vec{a_{c}}$ contains the output of the accelerometer sensor, and $\vec{g}$ represents the output of the gravity sensor.

(1) $$\vec{a}=\vec{a_{c}}-\vec{g}=\;\left(a_{x},a_{y},a_{z}\right),$$

After calculating $\vec{a}$ (which is referenced to the axes in Figure 1) we can project the acceleration component in the gravity direction (perpendicular to the Earth’s surface), which is independent of the relative position of the mobile device and the relative movements of the device while walking (when the device is carried not tight to the human body). Equation (2) captures the computation of the acceleration component in the gravity direction.

(2) $$\vec{a_{g}}=\frac{\vec{a}\cdot \vec{g}}{∥\vec{g}{∥}^{2}}\vec{g},$$

The value of $a_{g}=\vec{a_{g}}\cdot \frac{\vec{g}}{∥\vec{g}∥}$ provides the vertical acceleration while walking. In order to improve the detection accuracy, the acceleration information in the horizontal plane has also been used. In particular, we have used the $a_{Hx}$ component from Equation (3):

(3) $$\vec{a_{H}}=\vec{a}-\vec{a_{g}}=\left(a_{Hx},a_{Hy},a_{Hz}\right),$$

The raw data used as the input to the step detection algorithm will be the time sequence of $\left(a_{g},a_{Hx}\right)$, sampled at 50 Hz when the mobile device is carried in the user’s trouser pocket. Placing the sensor device close to the hip is a normal practice in step detection [5].

## 4. Outlier Pre-Detection

In order to save computation energy and optimize the search for candidate time segments that may contain a step, and at the same time provide an alignment for the time sequence, an outlier detection technique based on the Mahalanobis distance is proposed in this paper. Gravity compensated step related acceleration patterns have a prominent activity in the milliseconds after the ground contact of each foot. In order to detect steps of up to 60 steps per minute, a time window of up to 1 s should be used in order to detect outliers in the $\left(a_{g},a_{Hx}\right)$ sequence. Outliers are potential candidates that may contain a ground contact in a step.

In order to train the algorithm, a labeled sequence of steps at low speeds (at 30 and 40 steps per minute) from three different users sampled at 50 Hz is used. The outlier detection provides a set of candidate points that are post-filtered using the labeled information. The result is a set of outliers associated with ground contact instants of real steps executed at slow speeds. These steps will be used to compute the transition matrices and then train the final autoencoders (one for $a_{g}$ and another for $a_{Hx}$) for steps at slow speed detection.

## 5. Transition Matrices

The transition matrix computed from a time series captures the probability of moving from each state-a at instant “*t*” to each state-b in the next sample at “*t* + 1”. In our case, we have two acceleration time series $a_{g}\left(t\right),\;a_{Hx}\left(t\right)$. A transition matrix is computed for the acceleration samples around each pre-detected outlier. For each outlier at *t_i_*, the transition matrices are computed form $a_{g}\left(t_{i-N},\ldots ,t_{i},\ldots ,t_{i+N}\right)$ and $a_{Hx}\left(t_{i-N},\ldots ,t_{i},\ldots ,t_{i+N}\right)$. Each sample is assigned to a state according to the distance to the mean value of the series in terms of the standard deviation of the series. We have used eight states as follows (for the particular case of $a_{g}\left(t\right)$):

m = mean value of ($a_{g}\left(t_{i-N},\ldots ,t_{i},\ldots ,t_{i+N}\right)$)
std = standard deviation of $(a_{g}\left(t_{i-N},\ldots ,t_{i},\ldots ,t_{i+N})\right)$
S_1_ if $a_{g}\left(t\right)$ − m ≤ −1.5*std
S_2_ if $a_{g}\left(t\right)$ − m ≤ −std and $a_{g}\left(t\right)$ − m > −1.5*std
S_3_ if $a_{g}\left(t\right)$ − m ≤ −0.5*std and $a_{g}\left(t\right)$ −m > −std
S_4_ if $a_{g}\left(t\right)$ − m ≤ 0 and $a_{g}\left(t\right)$ − m > −0.5*std
S_5_ if $a_{g}\left(t\right)$ − m ≤ 0.5*std and $a_{g}\left(t\right)$ − m > 0
S_6_ if $a_{g}\left(t\right)$ − m ≤ std and *a_g_*(*t*) − m > 0.5*std
S_7_ if $a_{g}\left(t\right)$ − m ≤ 1.5*std and $a_{g}\left(t\right)$ − m > std
S_8_ if $a_{g}\left(t\right)$ − m > 1.5*std

The state vectors for outlier “*i*”, $s_{g}\left(t_{i-N},\ldots ,t_{i},\ldots ,t_{i+N}\right)$ and $s_{Hx}\left(t_{i-N},\ldots ,t_{i},\ldots ,t_{i+N}\right)$, are used to calculate the transition matrix at t_i_. The transition matrix counts the number of times that being at state j goes to state k in the next instant of time. A final regularization is performed to convert counts to probabilities by dividing the cumulative count of each row, as captured in Equation (4), where the component in row *j* and column k of the transition matrix is calculated.

(4) $$T\left(j,k\right)=\frac{Count\;\left(j\to k\right)}{\sum_{l}Count\;\left(j\to l\right)},$$

In order to use the transition matrices at each outlier as the input to the autoencoder, described in the next section, the matrix is converted into a vector by concatenating each row in the matrix one after the other.

## 6. Autoencoders

A method based on training two different autoencoders, one for the $s_{g}$ transition matrices at each step and a similar one based on $s_{Hx}$, transition matrices, have been used in order to detect steps from pre-selected outliers. The Pearson correlation index is used to calculate the similarity of the reconstructed transition matrix at each pre-selected outlier in the detection time series with the input one. If the Pearson correlation index for both autoencoders is above a certain threshold, than the pre-selected point is detected as a step. A description on how autoencoders work can be found in [25].

The design of an autoencoder tries to minimize the error between the reconstructed output and the input following Equation (5).

(5) $$\varepsilon \left(x,x\prime \right)=∥x-f_{2}{(W^{\prime }\left(f_{1}\left(Wx+b\right)\right)+b\prime )∥}^{2},$$

where *x*’ is the reconstructed signal after an encoder and decoder functions (*f*_1_ and *f*_2_ are activation functions such as the sigmoid function). In our case, *x* corresponds to the vector calculated from serializing the transition matrix *T*, as described in the previous section.

## 7. Results

This section presents the results of the conducted experiment. The set-up for the experiment is presented first (both in terms of the data gathered as well as the implemented algorithm details). A first sub-section is dedicated to present the experiment set-up and the database gathered. A second sub-section is dedicated to explaining the internal details of the implemented algorithm. Then, the obtained results are captured in two different sub-sections. A first sub-section shows the results achieved when directly applying the autoencoders to the acceleration time series around the pre-selected outliers. A second sub-section uses the transition matrices instead of the acceleration time series as the input to the final autoencoders in order to capture the time dependencies. The idea is not only to present the results of the proposed algorithm in terms of accuracy and recall, but also to assess the gain in these figures when using transition matrices instead of acceleration time series, as other previous research has presented.

As a mechanism to compare the detection results provided in each case, we have used the obtained precision, recall, and *F* score. If we define *tp* as the number of “steps walking at slow speeds” that are correctly detected, *Tp* as the total number of “steps walking at slow speeds” present in the validation sequence, and *fp* as the total number of “non-steps walking at slow speeds” that are detected as positive samples, the precision, the recall, and the *F* score can be defined, as shown in Equation (6).

(6) $$\begin{array}{l} \mathrm{precision}=\frac{\mathrm{tp}}{\left(\mathrm{tp}\;+\;\mathrm{fp}\right)}\\ \mathrm{recall}=\frac{\mathrm{tp}}{\mathrm{Tp}}\\ F=2\;\cdot \;\frac{\mathrm{precision}\cdot \mathrm{recall}}{\mathrm{precision}\;+\;\mathrm{recall}}, \end{array}$$

### 7.1. Experiment Set-up and Database

A group of three volunteers have recorded the output of both the acceleration and gravity sensors when wearing a Nexus 6 Android smart phone in the pocket of the trousers (close to the participant’s hip, a common place that has been widely used in previous literature for step detection and counting). The demographic details for the participants are captured on Table 1.

The implemented algorithm combining the techniques presented in Section 4, Section 5 and Section 6 should be trained with data that will characterize the objective class to be detected. For the case of this paper, the objective of the algorithm has been set to detect the steps executed at cadences between 30 and 40 steps per minute. Our objective is to maximize the number of true positives (the number of true steps detected) while minimizing the false positives (segments of other activities that are detected as members of the target class). Previous studies, such as [7], have shown how the reported number of steps by off-the-shelf devices tend to underestimate the real value for speeds around 67 steps per minute. In our research, we have designed an algorithm that could be trained to detect steps at even smaller cadences. Moreover, we have added the study of how the algorithm discriminates data from similar activities (which has been many times overlooked in previous studies such as [7]). We have generated a dataset that includes segments of data in the target class, as well as three other activities that may present similar acceleration patterns (sitting down and up, walking in circles at 30 steps per minute, and walking at 60 steps per minute). In order to generate the required data, each participant was asked to execute the following sequence of movements:

- to stand still for 5 s (this information will be used to validate the calibration of the gravity sensor and to mark the start of the data);
- walk at a speed of 60 steps per minute during 60 s;
- to stand still for 5 s (this information will be used to validate the calibration of the gravity sensor and facilitate the automatic split of the recorded data into segments of single activities);
- walk at a speed of 30 steps per minute during 60 s;
- to stand still for 5 s;
- walk at a speed of 40 steps per minute during 60 s;
- to stand still for 5 s;
- slide (walk without separating the feet from the ground) at a speed of 30 steps per minute during 60 s;
- to stand still for 5 s;
- sit down and up 10 times;
- to stand still for 5 s;
- walk around a chair (in circles) at a speed of 30 steps per minute during 60 s; and,
- to stand still for 5 s.

The movements contained in the database are all related to the target class of steps walking at speeds of 30 to 40 steps per minute. Walking without lifting the feet from the ground (sliding) at slow speeds is expected to generate similar $a_{Hx}\left(t\right)$ sequences than walking at the same speeds, but the algorithm should detect the differences in the $a_{g}\left(t\right)$ component. On the opposite site, sitting down and standing up could generate similar acceleration patterns in the gravity axis $a_{g}\left(t\right)$ but different ones in $a_{Hx}\left(t\right)$. Walking in circles around a chair will change the direction of the movement and so the acceleration components. Finally, walking at moderate speeds (60 steps per minute) will generate similar patterns for some of the steps. In the real application of counting steps for monitoring physical activities, all of the steps would count for a positive output. In the case of this research, we want to test the discrimination rate of the proposed algorithm for the particular case of walking at slow speeds from some similar classes.

### 7.2. Implemented Algorithm Details

The techniques presented in Section 4, Section 5 and Section 6 have been combined to implement two “steps at slow speeds” detection algorithms. This sub-section captures the design and implementation details for these algorithms. The experimental results for these algorithms using the data gathered as described in the previous sub-section are captured in Section 7.3 and Section 7.4.

The first algorithm will combine outlier detection techniques and autoencoders in order to detect “steps at slow speeds” from acceleration data. The algorithm details are:

- Define the maximum and minimum cadences of steps to be detected (c*_max_* and c*_min_*) in steps per minute → in our case c*_max_* = 40 and c*_min_* = 30
- Set the outlier detection window to $T_{out}=\frac{60}{c_{max}}s$
- For each T*_out_* = 1.5 s of $a_{g}\left(t\right),a_{Hx}\left(t\right)$ centered at $t_{c}$, calculate the Mahalanobis distance $\mathrm{md}\left(t_{c}\right)$ from $\left(a_{g}\left(t_{c}\right),a_{Hx}\left(t_{c}\right)\right)$ and $\left[\left(a_{g}\left(t_{c}-0.75\right),a_{Hx}\left(t_{c}-0.75\right)\right),\ldots ,\left(a_{g}\left(t_{c}+0.75\right),a_{Hx}\left(t_{c}+0.75\right)\right)\right]$
- For all $t_{c}$ in (0:$t_{max}$), if $\mathrm{md}\left(t=t_{c}\right)>\mathrm{th}$ then consider $t_{c}$ an outlier (th = 3 has been empirically selected).
- For each $t_{c}$. corresponding to an outlier use $\left[\left(a_{g}\left(t_{c}-0.12\right),a_{Hx}\left(t_{c}-0.12\right)\right),\ldots ,\left(a_{g}\left(t_{c}+0.12\right),a_{Hx}\left(t_{c}+0.12\right)\right)\right]$ to feed an autoencoder with a single hidden layer with 5 hidden units.
- Calculate the Pearson correlation index between the input and output of the autoencoder as a similarity index to decide if the outlier corresponds to a step.

The second algorithm will try to improve the results of the previous algorithm by characterizing the time sequences around each pre-detected outlier in terms of its transition matrix. The algorithm details are:

- Define the maximum and minimum cadences of steps to be detected (c*_max_* and *c_min_*) in steps per minute → in our case c*_max_* = 40 and c*_min_* = 30
- Set the outlier detection window to $T_{out}=\frac{60}{c_{max}}s$
- For each T*_out_* = 1.5 s of $a_{g}\left(t\right),a_{Hx}\left(t\right)$ centered at $t_{c}$, calculate the Mahalanobis distance $\mathrm{md}\left(t_{c}\right)$ from $\left(a_{g}\left(t_{c}\right),a_{Hx}\left(t_{c}\right)\right)$ and $\left[\left(a_{g}\left(t_{c}-0.75\right),a_{Hx}\left(t_{c}-0.75\right)\right),\ldots ,\left(a_{g}\left(t_{c}+0.75\right),a_{Hx}\left(t_{c}+0.75\right)\right)\right]$
- For all $t_{c}$ in (0:$t_{max}$), if $\mathrm{md}\left(t=t_{c}\right)>\mathrm{th}$ then consider $t_{c}$ an outlier (th = 3 has been empirically selected).
- For each $t_{c}$ corresponding to an outlier use $\left[\left(a_{g}\left(t_{c}-0.12\right),a_{Hx}\left(t_{c}-0.12\right)\right),\ldots ,\left(a_{g}\left(t_{c}+0.12\right),a_{Hx}\left(t_{c}+0.12\right)\right)\right]$ in order to estimate the transition matrix as described in Section 5 (being N = 6). The values of $a_{g}\left(t\right)\;\mathrm{and}\;a_{Hx}\left(t\right)$ are mapped into 8 different states (this number has been empirically selected) as described in Section 5. The states are assigned depending on the distance of each pair $a_{g}\left(t\right)$, $a_{Hx}\left(t\right)$ to the mean values of $a_{g}\left[T\right]$ and $a_{Hx}\left[T\right]$ in the previously selected 240 ms time window centered at each outlier in terms of their standard deviation. This normalization is required in order to compensate different user weights.
- Use the transition matrices to feed an autoencoder with a single hidden layer with five hidden units.
- Calculate the Pearson correlation index between the input and output of the autoencoder as a similarity index to decide if the outlier corresponds to a step.

Both of the algorithms have been implemented in Matlab. The mahal function has been used in order to calculate the Mahalanobis distance. The trainAutoencoder [26], encode and decode functions have been used for the autoencoder.

### 7.3. Autoencoders Based on Acceleration Data around Outlier Pre-Detected Points

The data from the segments of slow walking (30 and 40 steps per minute) from two out of the three participants were isolated in order to train the autoencoders. Each step was detected by using the outlier pre-detection phase previously described in Section 4. The time location of each detected outlier was compared with a predefined vector manually introduced containing the ground truth (manual labels) for each step. The outlier pre-detection phase was needed in order to automatically align the segments of the acceleration data associated with each step (the manual labeling process did not require therefore the exact location of the ground contact instant but a visual approximation, eliminating therefore the human error when labeling the data). Only two participants have been selected for training to implement a leave one out validation approach.

**Figure 2:**
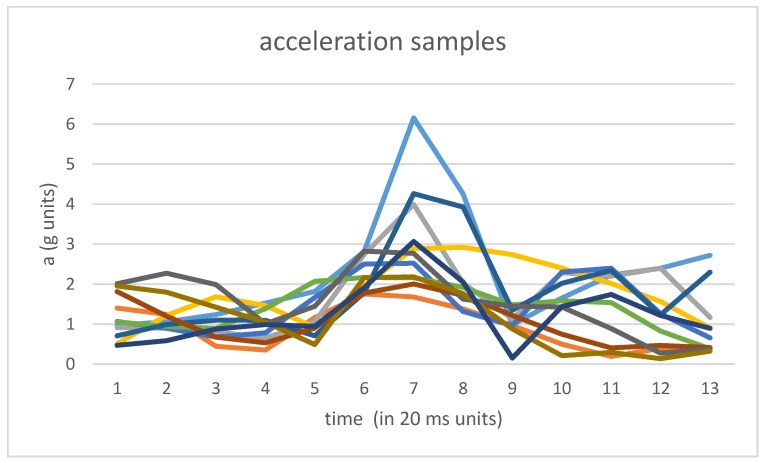
Acceleration samples around outliers corresponding to slow walking steps. Each color represents a different sample.

In order to generate the samples of acceleration, windows of 240 ms of raw data (both accelerometer and gravity data) have been used around each pre-detected outlier corresponding to each labeled step. The size of 240 ms. has been empirically selected in order to include all of the acceleration patterns corresponding to the ground contact instant. Figure 2 captures some of the acceleration samples a(t) corresponding to outliers associated with labeled steps.

For each window of pre-selected data, the $a_{g}\left(t\right),\;a_{Hx}\left(t\right)$ components were computed. The samples of each component were used to train a different autoencoder.

After finishing with the training of the autoencoders, the validation phase used all of the recorded data from the third participant in order to detect steps (the leave-one-out process was repeated with all tree participants). Windows of acceleration data were computed for each pre-detected outlier in a similar way, but now for the entire time series containing all of the movements for each of the three participants. A Pearson correlation index was used to compare the output and the input of the autoencoders and different detection thresholds have been used to assess the obtained results. The idea is that the acceleration sequences computed for similar steps to those used in the training of the algorithm will show high values for the Pearson correlation index. However, acceleration sequences computed for other movements from those used in the training of the algorithm will show small values for the Pearson correlation index.

The results obtained for the recall, precision, and *F*-score for different similarity thresholds are captured in Table 2. The optimal value for the *F*-score is achieved when using a similarity threshold of 0.6. The recall in this case is 0.67 and the precision is 0.59. A recall = 0.67 means that we are able to detect 67% of all steps executed at slow speeds (30 and 40 steps per minute). A precision = 0.59 means that we detect 41% of false positives (other activities detected as slow walking steps). The recall of the algorithm could be improved by detecting slow walking segments in which missing intermediate walking steps could be incorporated into the output of the algorithm. The idea is to estimate the walking cadence as the inverse of the difference in time between two consecutive detected steps. When the cadence shows a relatively small variation among consecutive steps for the last steps and the time between two consecutive detected steps suddenly approximates the double of previous steps, we could estimate that one more step has been executed. In our case, we have not used this detection feature since we only had a limited number of walking segments and the results will be close to the optimal value of recall = 1 since it is known that the test person did not perform intermediate stops (except for the steps missing at the beginning of a waking segment).

The average distribution of false positives among the different movements is captured in Table 3. No outlier in the acceleration time series for the sit down and stand up segments is classified as walking at slow speeds. The horizontal acceleration component is different and there is no confusion for this movement. Only a moderate 6.83% of walking in circles steps are detected as walking at slow speeds. The horizontal component is again different. The majority of false positives are related to sliding sections. In this case, the horizontal component is similar to the target class and some of the misclassified segments show a similar vertical acceleration pattern despite the fact that the foot is not separated from the ground (the sensor is located in the pocket of the participant which captures some vertical acceleration activity). Finally, some of the walking at moderate speed steps are also generating false positives and showing similar patterns as those steps executed at slower speeds.

### 7.4. Autoencoders Based on Transition Matrices around Outlier Pre-Detected Points.

In this sub-section, the same computations are performed but using the transition matrices instead of the acceleration time series in order to train the autoencoders and perform step detection. Transition matrices capture the temporal dependencies from adjacent samples in acceleration segments. We use the method proposed in Section 5 to compute the transition matrices and to convert them into vectors in order to feed the final autoencoders.

The results obtained in this case for the recall, precision, and *F*-score for different similarity thresholds are captured in Table 4. The recall, precision, and *F* scores improve as compared to the previous section. For a value of the similarity threshold of 0.4, a recall of 0.88 is achieved, meaning that 88% of the steps walking at slow speeds are counted (with 50% of false positives among the related movements). The optimal value for the *F*-score is achieved when using a similarity threshold of 0.8. The optimal *F*-score is in this case 0.67, improving from 0.63 in the case of using acceleration time series instead of transition matrices. A balanced result for both recall and precision is achieved for a similarity threshold of 0.7, in which approximately 2/3 of the steps walking at slow speeds are detected (without using the post-detection estimation of missing steps previously described) and 2/3 of true positives are detected (1/3 of false positives).

**Figure 3:**
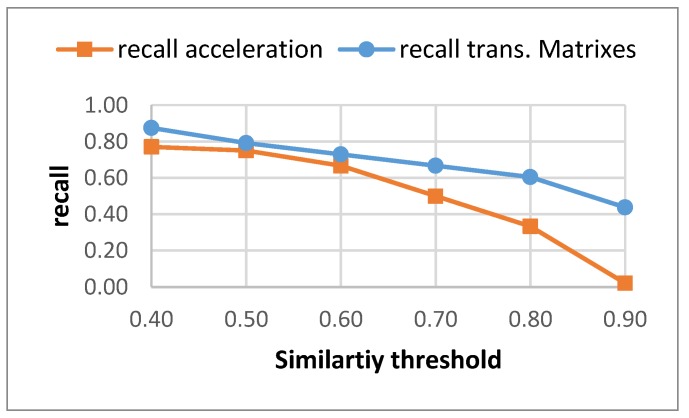
Recall for different similarity threshold for both methods.

Figure 3 captures a visual comparison for the recall achieved by both methods for different similarity thresholds. Using the transition matrices to characterize the time dependencies improves the results obtained for all of the similarity threshold values. The results do not decay so fast when the threshold moves close to 1.

**Figure 4:**
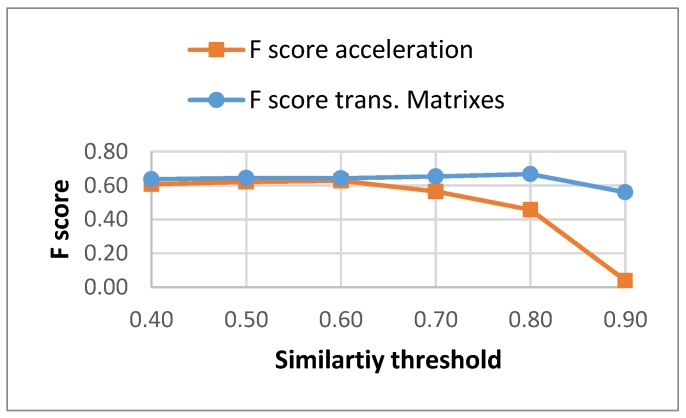
*F*-score for different similarity threshold for both methods.

The results for the *F*-score for the different similarity thresholds for both methods are captured in Figure 4. Using the transition matrices instead of time acceleration segments generates a more constant *F*-score along the different similarity thresholds (the lost in recall is compensated by a similar gain in precision).

The average distribution of false positives among the different movements for the case of using transition matrices to capture the time dependencies is shown in Table 5. Similar results to those presented in Table 3 are obtained. In this case, there is a small decrement in the percentage of steps at 60 steps per minute detected as walking at slower speeds, which is compensated by the increment in slide segments detected, as walking at slow speeds.

## 8. Conclusions

This study has proposed and validated a new method to detect steps while walking at slow speeds. The algorithm combines the use of outlier detection in acceleration time series, sensor random movements compensation, time dependencies modelling in acceleration series, and a final detection phase. We have used the Mahalanobis distance to detect outliers in the time series. A threshold has been used so that the ground contact instant in steps walking at slow speeds is detected as an outlier in at least 95% of the recorded data. Random movements of the sensor when not tightly worn to the participant’s body and initial placement compensation has been conducted by using the gravity sensor to estimate the direction of the gravity force. Temporal dependencies have been modeled using transition matrices. A comparison when acceleration time series are used instead of transition matrices is also presented in the paper. Finally, we have used autoencoders and a similarity function based on the Pearson correlation index in order to perform the final step detection.

The results have been validated using a new database that we have recorded using three participants executing a series of movements that are similar in terms of acceleration patterns to the target class that we want to detect (walking at slow speeds). Using acceleration time series as the input to the final autoencoders achieves an optimal *F* score of 0.63 for the data in the recorded database. Using transition matrices to model the time dependencies improves the results to a *F* score value of 0.67.

Similar movements have been introduced in the recorded database to validate the proposed algorithm in a pessimistic scenario. The results show that around half of the false positives are for steps executed at higher speeds, which show similar patterns around the ground contact instant to steps at slower speeds. These false positives will contribute to positive results for the generic approach of detecting steps while walking at any speed in applications to motivate the physical exercise to people in need.

As a future work, the study will include other parts of the body to which to attach the accelerometer sensor and using devices from different manufacturers in order to generalize results. Moreover, participants suffering long-term conditions, such as COPD, will also be added in order to validate the applicability of the proposed algorithms for different types of users.

## Figures and Tables

**Table 1:** Participant demographics.

Participant ID	Age	Gender	Normal Walk
1	24	M	Y
2	41	F	Y
3	45	M	Y

**Table 2:** Results for different similarity thresholds.

Sim Thr	Recall	Precision	*F* Score
0.40	0.77	0.50	0.61
0.50	0.75	0.53	0.62
0.60	0.67	0.59	0.63
0.70	0.50	0.65	0.56
0.80	0.33	0.73	0.46
0.90	0.02	0.50	0.04

**Table 3:** Percentage of false positives.

	Sit down	Walk in Circles	Slide	Walk 60 spm
% detected as →	0.00	6.83	55.82	37.36

**Table 4:** Results for different similarity thresholds.

Sim Thr	Recall	Precision	*F* Score
0.40	0.88	0.50	0.64
0.50	0.79	0.54	0.64
0.60	0.73	0.57	0.64
0.70	0.67	0.64	0.65
0.80	0.60	0.74	0.67
0.90	0.44	0.78	0.56

**Table 5:** Percentage of false positives.

	Sit down	Walk in Circles	Slide	Walk 60 spm
% detected as →	0.00	5.07	46.20	48.73
